# Recurrence in oral and pharyngeal cancer is associated with quantitative *MGMT *promoter methylation

**DOI:** 10.1186/1471-2407-9-354

**Published:** 2009-10-06

**Authors:** Emanuela Taioli, Camille Ragin, Xiao-hong Wang, Jiangying Chen, Scott M Langevin, Ashley R Brown, Susanne M Gollin, Seymour Garte, Robert W Sobol

**Affiliations:** 1 University of Pittsburgh Cancer Institute, Hillman Cancer Center, Pittsburgh, PA, USA; 2 Department of Pharmacology & Chemical Biology, University of Pittsburgh School of Medicine, Pittsburgh, PA, USA; 3 Department of Human Genetics, University of Pittsburgh Graduate School of Public Health, Pittsburgh, PA, USA; 4 Department of Epidemiology, University of Pittsburgh graduate School of Public Health, Pittsburgh, PA, USA; 5 Department of Environmental and Occupational Health, University of Pittsburgh Graduate School of Public Health, Pittsburgh, PA, USA; 6 SUNY Downstate School of Public Health, Graduate Program in Public Health at SUNY Downstate Medical Center, Brooklyn, NY, USA

## Abstract

**Background:**

Biomarkers that predict clinical response, tumor recurrence or patient survival are severely lacking for most cancers, particularly for oral and pharyngeal cancer. This study examines whether gene-promoter methylation of tumor DNA correlates with survival and recurrence rates in a population of patients with oral or pharyngeal cancer.

**Methods:**

The promoter methylation status of the DNA repair gene *MGMT *and the tumor suppressor genes *CDKN2A and RASSF1 *were evaluated by methylation-specific PCR in 88 primary oral and pharyngeal tumors and correlated with survival and tumor recurrence. Quantitative *MGMT *methylation was also assessed.

**Results:**

29.6% of the tumors presented with *MGMT *methylation, 11.5% with *CDKN2A *methylation and 12.1% with *RASSF1 *methylation. *MGMT *promoter methylation was significantly associated with poorer overall and disease-free survival. No differences in methylation status of *MGMT *and *RASSF1 *with HPV infection, smoking or drinking habits were observed. A significant inverse trend with the amount of *MGMT *methylation and overall and disease-free survival was observed (p_trend _= 0.002 and 0.001 respectively).

**Conclusion:**

These results implicate *MGMT *promoter methylation as a possible biomarker for oral and pharyngeal cancer prognosis. The critical role of MGMT in DNA repair suggests that defective DNA repair may be correlative in the observed association between *MGMT *promoter methylation and tumor recurrence. Follow-up studies should include further quantitative MSP-PCR measurement, global methylation profiling and detailed analysis of downstream DNA repair genes regulated by promoter methylation.

## Background

Oral and pharyngeal squamous cell carcinoma is the most common malignant neoplasm of the head and neck region [1]. An estimated 34,360 new patients were diagnosed with squamous cell carcinoma of the head and neck and 7,550 deaths occurred in the U.S. in 2007, accounting for 2-3% of all malignancies [2]. About 30-50% of oral and pharyngeal cancer patients will develop a recurrence and/or a second primary tumor within two decades of the initial diagnosis. The five-year survival rate for oral cavity cancer has changed little between 1975 and 2002, approximately 48% [2]. Despite the progress made in early detection and therapy, early predictors of cancer recurrence at time of diagnosis are still missing for oral and pharyngeal cancer.

Epigenetic changes in cancer have traditionally been evaluated by measuring the status of CpG island cytosine methylation of a particular gene, such as *MGMT *[3]. It has been well-documented in cell lines, xenografts and in clinical trials, that methylation at discrete regions within CpG islands of a given gene promoter results in gene silencing and therefore prevents expression of the corresponding gene [3]. Several genes (oncogenes, tumor suppressor genes, DNA repair genes and growth control genes) have been shown to have an aberrant methylation profile (promoter hypermethylation) in tumors as compared to normal tissue or blood cells or cells in bodily fluids [4-6]. The altered DNA methylation landscape of the cancer epigenome is not limited to promoter hypermethylation of select genes, but also includes global hypomethylation as a prelude to oncogene activation and genome instability [7-9].

It has been suggested that aberrant methylation patterns could therefore act as a selective factor on neoplastic cells, influencing patients' survival and prognosis, particularly if methylation affects expression of a tumor suppressor gene such as *CDKN2A*, *RASSF1 *or a DNA repair gene such as *MGMT*. For example, aberrant gene hypermethylation of *CDKN2A*, *p14ARF *[10], *RASSF1 *[11-13] and the DNA repair gene *MGMT *[3] have been reported in tumor tissue of oral cavity cancer patients (see Table 1 for a more comprehensive list of genes methylated in oral cancers). In addition, promoter methylation of *CDKN2A*, *MGMT*, *DAPK1*, and *CDH1 *has been studied in relation to head and neck cancer survival [14-17]. However, the role of hypermethylation on outcome in oral and pharyngeal cancer patients, a special subset of head and neck cancers, has not yet been addressed.

**Table 1:** Promoter methylation of genes in Oral cancer

Gene	Cancer type	Method for methylation analysis	Reference
*TSC2*	Oral squamous cell carcinoma (OSCC)	COBRA	[44]

*CDKN2A, p14ARF*	OSCC	MSP	[10]

*MGMT*	Head & Neck squamous cell carcinoma (HNSCC)	MSP	[3]

*RASSF1A*	Betal-associated oral carcinoma	MSP	[13]

*p16INK4a, p15, MLH1, MGMT, E-CADHERIN*	OSCC	Restriction multiplex PCR	[29]

*p16INK4a, RASSF1A, DAPK*	Salivary adenoid cystic carcinoma	MSP	[30]

*MGMT*	HNSCC	MSP	[21]

*p16INK4a*	HNSCC	MSP	[22]

*MGMT, p16INK4a, MLH1*	HNSCC	MSP	[23]

*CNKN2, CDH1, MGMT, DAPK, DBC1, p14, CDKN2B, RARB, RASSF1A, MLH1, p73, DCC, FHIT, SERPINB5*	HNSCC &/or OSCC	MSP, PCR-based restriction assay and/or bisulfite sequencing	[11]

*p16INK4a, CYCLIN A1, RARB, E-CADHERIN, MGMT, STAT1, ATM, MLH1, TIMP3*	SCC of the oral cavity or oropharynx	pryosequencing	[26]

*RASSF2*	OSCC	MSP	[45]

*RECK*	OSCC	MSP	[46]

*ATM*	HNSCC	MSP	[47]

*p16INK4a, CYTOGLOBIN, CYCLIN A1*	OSCC	pyrosequencing	[35]

*MLH1*	HNSCC	MSP	[15]

*p16INK4a, MGMT, DAPK, E-CADHERIN*	Laryngeal and hypopharyngeal cancer	MSP	[24]

*CYTOGLOBIN*	Oral or oropharyngeal squamous cell carcinoma	pyrosequencing	[48]

*RIZ1*	Nasopharyngeal carcinoma	MSP	[49]

*CDKN2A*	OSCC		[50]

*p16INK4a, p14ARF, MGMT, RB1, PTEN, p27KIP1*	OSCC	MSP	[51]

*APC*	OSCC	MSP	[52]

*CDH1*	HNSCC	MSP	[53]

*p16INK4a, DAPK, E-CADHERIN, RASSF1A*	HNSCC	MSP	[54]

*TIMP3, CDH1*	HNSCC	MSP	[55]

*p16INK4a*	Oral epithelial dysplasia	MSP	[56]

*RARB, MGMT, RASSF1, E-CADHERIN*	Salivary gland carcinoma	pyrosequencing	[57]

*MGMT, p16INK4a*	OSCC	MSP	[31]

*RASSF1A, RASSF2A, HIN-1*	OSCC	PCR-denaturing HPLC	[12]

*MLH1, MSH2*	OSCC	MSP	[58]

*RUNX3*	OSCC	MSP	[59]

*LINE-1*	OSCC	COBRALINE-1	[60]

*SFRP1, SFRP2, SFRP5*	OSCC	MSP	[61]

*miR-137*	OSCC	COBRA and bisulfite sequencing	[62]

Oral and pharyngeal cancers are for the large part squamous cell histological types, and are often anatomically grouped with head and neck cancers. Although the head and neck tumors have been historically grouped together due to similar etiology, the oral cavity, pharynx and larynx are unique structures with different functions and possibly different sensitivities to carcinogens, especially alcohol and tobacco. Human Papillomavirus is another etiologic agent involved in oral and pharyngeal tumors [18,19]. In the study described herein, we tested the association between promoter methylation and survival in a cohort of 88 oral and pharyngeal cancer patients, focusing on three target genes: the DNA repair gene *MGMT *and the tumor suppressor genes *CDKN2A and RASSF1*.

## Methods

### Study population

A database of head and neck cancer cases was established in June 2004 at the University of Pittsburgh Medical Center [19] for patients undergoing surgical resection of their tumors between November 1992 and February 1997. Demographics, smoking and alcohol use, family history, tumor site, clinical characteristics of the initial primary tumor and subsequent tumors, follow-up data (such as disease outcome and time to next disease occurrence) through June 2007 were obtained from the University of Pittsburgh Tumor Registry and from the review of clinical charts. Patients' clinical and demographic information was re-verified from each patient's original de-identified and coded questionnaires, pathology and surgical reports. This database contains clinical, treatment and follow-up information for the first surgical resection at the time of enrollment and all subsequent resections for all patients enrolled in the study. Tumor site, histology, stage and grade were classified according to the American Joint Committee on Cancer (AJCC) ICD9 codes, ICD-morphology, stage and grade classification, respectively. Samples were collected under IRB approval of the University of Pittsburgh Head and Neck SPORE tissue bank. All tumors samples were consented by the SPORE tissue bank and IRB approval is for data analysis as described herein.

Information on 88 subjects undergoing surgical resection of their first primary tumors was selected from the original database; HPV status was determined for all the tumors by PCR as previously described [19]. Sites of the oral cavity included cheek, retromolar area, alveolar ridge, oral tongue, palate, floor of mouth and overlapping lesions of other and unspecified parts of the mouth. The oropharynx included sites involving the base of tongue, soft palate, tonsil and overlapping lesions of the oral cavity and pharynx.

### Methylation-specific PCR

Previously extracted DNA from tumors was utilized in this study. DNA was isolated from primary fresh-frozen tumor tissue by guanidine thiocyanate extraction using the commercially available IsoQuick kit (MicroProbe, Garden Grove, CA) as we have described previously [20]. Methylation-specific PCR was used for the analysis of *MGMT, RASSF1 *and *CDKN2A *promoter methylation as described [3,21-24] using the Zymo Research EZ DNA Methylation-Gold reagents (ZYMO Research) according to the manufacturer's instructions. The primers used to assess *MGMT *promoter methylation status were: Methylated MGMT allele - tttcgacgttcgtaggttttcgc and gcactcttccgaaaacgaaacg; annealing temperature = 58°C; expected amplicon size = 81 bp. Un-methylated MGMT allele - tttgtgttttgatgtttgtaggtttttgt and aactccacactcttccaaaaacaaaaca; annealing temperature = 58°C; expected amplicon size = 93 bp [3]. The primer sequences to assess *RASSF1 and CDKN2A *methylation status were previously described [17,25]. A DNA methylated control using either Methylated or Unmethylated DNA (Chemicon; Millipore) is included in all the DNA modification and PCR reactions. PCR reaction products were then separated on 4% agarose gels containing ethidium bromide with 100 bp DNA Ladder Markers (Bioline) and examined under ultraviolet illumination to identify the distinct bands. Each PCR reaction was run in duplicate.

### Quantitative Methylation Analysis

To determine the degree of methylation among subjects found to have *MGMT *hypermethylation by MSP, quantitative methylation analysis was performed using a pyrosequencing methylation assay [26,27]. DNA samples that yielded a positive result in the MSP assay were then subjected to a quantitative methylation test using the PyroMark MGMT ID system, as per the manufacturer's instructions (Biotage, Inc.). Briefly, DNA was bisulfite treated using the Zymo Research EZ DNA Methylation-Gold reagents as indicated above. The treated DNA was amplified by PCR with MGMT-specific primers and twenty microliters of the amplicon was then subjected to pyrosequencing using the Biotage PyroMark Q24 System. CpG site quantification was performed with the Biotage methylation Software PyroQ-CpG™. The average percent methylation for MGMT was calculated from direct measurement of the percentage of methylation at five individual CpG sites.

### Statistical analyses

Follow-up, demographic, clinical and laboratory data for the study population was extracted from the head and neck database and imported to a statistical software package for analysis. Statistical analyses were performed using the Intercooled STATA (version 8.2) software (StataCorp. LP, College Station TX). Vital status and recurrence were the primary statistical endpoints for survival. Overall survival was defined as the time period between the surgical resection of the initial primary tumor and death. Disease-free survival was defined as the time period between the surgical resection of the initial primary and tumor recurrence. All patients lost to follow-up were censored. Kaplan-Meier survivor functions for overall and disease-free survival were generated using STATA. The log-rank statistic was used to test the equality of survivor functions. Multivariable Cox proportional hazards models were created for overall and disease-free survival for both MSP data for *CDKN2A*, *RASSF1A*, and *MGMT*; and quantitative *MGMT *methylation data generated from the pyrosequencing assay. For the quantitative data models, degree of *MGMT *methylation status was classified as unmethylated, methylation index ≤ 6.9%, and methylation index > 6.9%, based on the median of the whole population, and was analyzed as a categorical variable. Age, race, gender, smoking status, alcohol consumption, treatment and stage at diagnosis were considered for inclusion in the model. All covariates with P < 0.25 were retained in the final model.

## Results

### Patient demographics

The description of the population under study is reported in Table 2. As expected from the epidemiology of oral and pharyngeal cancer, there was a 2:1 male:female ratio. The majority of the subjects were ever smokers (87.5%) and ever drinkers (81.8%). The average follow up was 65.8 ± 48.9 months. During the follow-up, 42.0% of the patients developed a recurrence, and 53.4% died. More than half of the patients (59%) were in stage III or IV at diagnosis; half of the patients were treated with surgery alone, half with a combination of surgery and radiotherapy and/or chemotherapy.

**Table 2:** Description of the study population

Characteristic	Study Population n (%)
Age (years), mean ± std dev	62.2 ± 13.0
Gender	
Male	56 (63.6%)
Female	32 (36.4%)

**Smoking status**	
Never smoker	11 (12.5%)
Ever smoker	77 (87.5%)
Alcohol Use	
Never drinker	16 (18.2%)
Ever drinker	72 (81.8%)

**Family history**	
Negative	41 (46.6%)
Positive	47 (53.4%)

**Histology**	
Squamous	85 (96.6%)
Adenoid Cystic	1 (1.1%)
Mucoepidermoid	2 (2.3%)

**Anatomic site**	
Oral cavity	50 (56.8%)
Oropharynx	38 (43.2%)

**Stage at diagnosis**	
I	20 (22.7%)
II	16 (18.2%)
III	17 (19.3%)
IV	35 (39.8%)

**Treatment**	
Surgery only	44 (50.0%)
Surgery and radiotherapy	39 (44.3%)
Surgery and chemoradiation	5 (5.7%)

### MGMT, CDKN2A and RASSF1 methylation status and outcome

**Figure 1:**
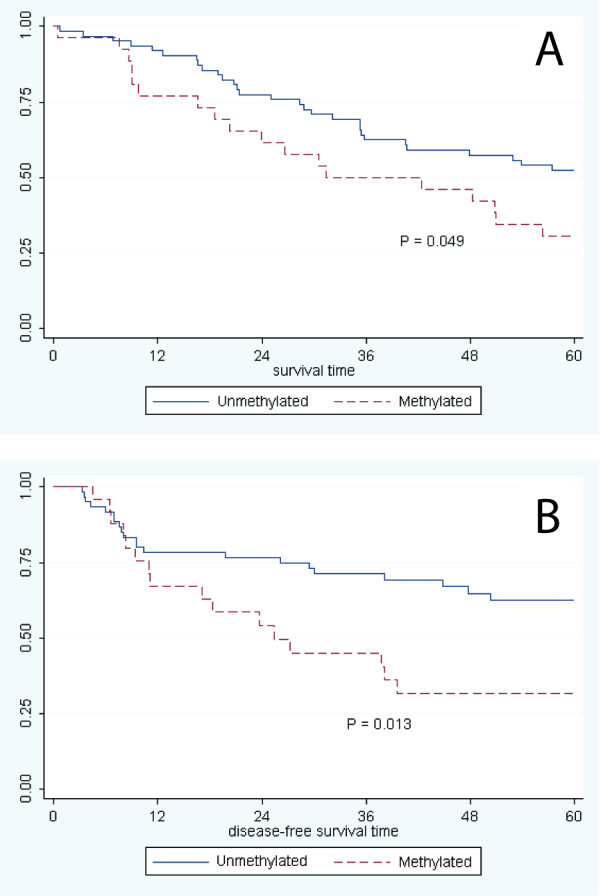
**MGMT promoter methylation status**. Methylation at the MGMT promoter was determined by methylation specific PCR, followed by gel electrophoresis. Shown are the plots of **(A) **overall patient survival time (months) and **(B) **disease-free survival time (months), according to *MGMT *methylation status (unmethylated or methylated).

Promoter methylation at *MGMT *was seen in 29.6% (26/88) of the tumors, 11.5% (7/61) showed *CDKN2A *methylation and 12.1% (8/66) *RASSF1 *methylation. The distribution of methylation status in the three genes studied according to several personal and clinical characteristics is presented in Table 3. Methylation of *CDKN2A *was significantly more frequent in never drinkers, and in less severe stages at diagnosis. Conversely, no association was observed between methylation status of *MGMT*, or *RASSF1 *and any of the variables analyzed, including smoking or drinking habits, stage at diagnosis or family history of cancer. *MGMT *promoter methylation was significantly inversely associated with overall survival (p = 0.049; Figure 1A) and disease-free survival (p = 0.013; Figure 1B). A non-significant association of promoter hypermethylation and both recurrence and survival was observed for *CDKN2A *(data not shown).

**Table 3:** Description of methylation status by methylation-specific PCR (MSP) in relation to main demographic and clinical parameters

	*MGMT *Methylation (%)	*RASSF1 *methylation (%)	*CDKN2A *methylation (%)
**Gender**			
Male	14/56 (25.0%)	4/44 (9.1%)	5/40 (12.5%)
Female	12/32 (37.5%)	4/22 (18.2%)	2/21 (9.5%)

**Smoking status**			
Never smoker	5/11 (45.5%)	0/9 (0.0%)	2/8 (25.0%)
Ever smoker	21/77 (27.3%)	8/57 (14.0%)	5/53 (9.4%)

**Alcohol use**			
Never drinker	4/16 (25.0%)	0/12 (0.0%)	4/12 (33.3%)*
Ever drinker	22/72 (30.6%)	8/54 (14.8%)	3/49 (6.1%)*

**Family history**			
Negative	11/41 (26.8%)	3/35 (8.6%)	4/30 (13.3%)
Positive	15/47 (31.9%)	5/31 (16.1%)	3/31 (9.7%)

**Histology**			
Squamous	24/85 (28.2%)	8/65 (12.3%)	7/60 (11.7%)
Other	2/3 (66.7%)	0/1 (0.0%)	0/1 (0.0%)

**Anatomic site**			
Oral cavity & lip	12/50 (24.0%)	5/37 (13.5%)	6/35 (17.1%)
Oropharynx	14/38 (36.8%)	3/29 (10.3%)	1/26 (3.8%)

**Stage at diagnosis**			
Local (I, II)	11/36 (30.6%)	5/27 (18.5%)	4/26 (15.4%)*
Advanced (III, IV)	15/52 (28.8%)	3/39 (7.7%)	3/35 (8.6%)*

**Treatment**			
Surgery only	9/44 (20.5%)	3/26 (11.5%)	3/24 (12.5%)
Surgery and radiotherapy	16/39 (41.0%)	5/40 (12.5%)	4/37 (10.8%)
Surgery and chemoradiation	1/5 (20.0%)	-----	-----

**HPV**			
Negative	18/67 (26.9%)	7/47 (14.9%)	7/45 (15.6%)
Positive	8/21 (38.1%)	1/19 (5.3%)	0/7 (0.0%)

* Statistically significant (α = 0.05)

In the multivariate Cox models, *MGMT *hypermethylation was significantly inversely associated with overall survival (HR = 2.17, 95% CI: 1.11-4.23), after adjustment for age and stage at diagnosis, smoking, alcohol consumption; and disease-free survival (HR = 3.49, 95% CI: 1.62-7.52), adjusting for age and stage at diagnosis, smoking, alcohol consumption, treatment. There was no association between hypermethylation of *CDKN2A *or *RASSF1 *and overall or disease-free survival (Table 4).

**Table 4:** Multivariate Cox models for the association between gene methylation and cancer survival/cancer recurrence

		Hazard Ratio	
	*MGMT*	*CDKN2A*	*RASSF1*
**Overall Survival**			
Unmethylated	1 (reference)	1 (reference)	1 (reference)
Methylated	2.17 (1.11-4.23)^A^	1.41 (0.35-5.75)^C^	0.88 (0.20-3.84)^C^
< 6.9% Methylated	1.52 (0.59-3.91)^B^	not evaluated	not evaluated
≥ 6.9% Methylated	4.38 (1.78-10.76)^B^	not evaluated	not evaluated
Test of trend	p = 0.002	---	---
**Disease-Free Survival**			
Unmethylated	1 (reference)	1 (reference)	1 (reference)
Methylated	3.49 (1.62-7.52)^D^	2.57 (0.47-14.12)^C^	2.54 (0.82-7.93)^C^
< 6.9% Methylated	3.03 (1.24-7.44)^B^	not evaluated	not evaluated
≥ 6.9% Methylated	5.46 (1.75-17.00)^B^	not evaluated	not evaluated
Test of trend	p = 0.001	---	---

^A ^Adjusted for age, smoking, alcohol use, and stage at diagnosis

^B ^Adjusted for age, gender, alcohol use, and stage at diagnosis

^C ^Adjusted for age, alcohol use, and stage at diagnosis

^D ^Adjusted for age, smoking, alcohol use, treatment, and stage at diagnosis

For a subset of the population (n = 61) information on methylation status of all three genes was available (Table 5). Only 1.6% of the samples showed simultaneous hypermethylation of *MGMT*, *CDKN2A*, and *RASSF1*, while 8.2% of the samples reported methylation in two of the investigated genes. These frequencies do not diverge significantly from expected frequencies derived from a random combination of methylated genes.

**Table 5:** Proportion of promoter hypermethylation in *MGMT, CDKN2A *and *RASFF1 *in oral and pharyngeal cancer tissues

N of subjects (%) N = 61	*MGMT*	*CDKN2A*	*RASSF1*
35 (57.3)	-	-	-
13 (21.3)	+	-	-
5 (8.2)	-	+	-
2 (3.3)	-	-	+
4 (6.6)	+	-	+
1 (1.6)	+	+	-
0 (0.0)	-	+	+
1 (1.6)	+	+	+

+: methylated

-: unmethylated

All of the HPV-positive tumors carried non-methylated *CDKN2A *promoters, while no differences in methylation status of *MGMT *and *RASSF1 *with HPV infection was observed.

### Methylation status in peripheral blood

No *RASSF1 *or *CDKN2A *methylation was observed in DNA extracted from peripheral blood lymphocytes, while one sample (1.3%), a T4N0 M0 (stage IVa) squamous cancer of the oral cavity, showed *MGMT *methylation in the DNA sample obtained from blood cells.

### Quantitative methylation analysis of MGMT

**Figure 2:**
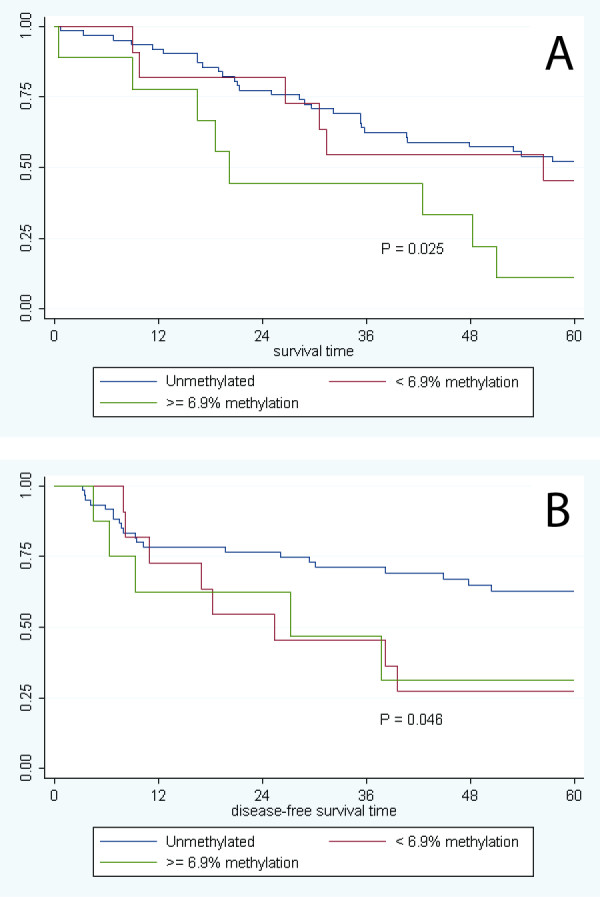
**Degree of MGMT promoter methylation**. The degree of methylation at the MGMT promoter was determined by pyrosequencing (Pyromark MGMT ID system). Shown are the plots of **(A) **overall patient survival time (months) and **(B) **disease-free survival time (months), according to the degree of *MGMT *methylation (no detectable methylation or a level of methylation either greater than or less than 6.9%).

We were able to obtain pyrosequencing results for 20/26 samples that tested positive for MGMT promoter methylation by MSP (3 samples had no remaining DNA; and 3 resulted as "not determined"). The median MtI value was 6.9% methylation, which ranged from 0.6% to 52.6%. Patients with high levels of *MGMT *promoter methylation (> 6.9%; n = 10) showed an overall significant decreased survival relative to patients with no *MGMT *hypermethylation (HR = 4.38, 95% CI: 1.78-3.91), after adjustment for age, gender, alcohol consumption, and stage at diagnosis. There was no association between the presence of less than or equal to 6.9% methylation (n = 10) and overall survival (HR = 1.52, 95% CI: 0.59-3.91). A significant inverse trend in survival with the amount of *MGMT *methylation was observed (p_trend _= 0.002; Table 4). Similar differences were observed in the Kaplan-Meier survival function (p = 0.025; Figure 2A).

The degree of *MGMT *hypermethylation was also associated with poorer disease-free survival (p_trend _= 0.001). Those with the highest degree of *MGMT *hypermethylation experienced poorer disease-free survival (HR = 5.46, 95% CI: 1.75-17.00) than patients with ≤ 6.9% *MGMT *methylation (HR = 3.03, 95% CI: 1.24-7.44) relative to subjects with no *MGMT *hypermethylation. A positive inverse trend was observed between the degree of *MGMT *methylation and disease-free survival (p_trend _= 0.001). These results were observed in the unadjusted Kaplan-Meier survival function as well (p = 0.046; Figure 2B).

## Discussion

Current research has shown that a number of tumor suppressor genes may be inactivated not only by genetic mechanisms such as deletions or point mutations, but also by hypermethylation or other similar epigenetic mechanisms [28] (see Table 1). While it is unclear whether environmental factors are responsible for gene hypermethylation, it has become clear that the presence of hypermethylation could be one of the predictors of prognosis [14-17,29,30]. We have analyzed here promoter methylation in three genes that have been involved in head and neck cancer prognosis, but focused our attention on the predictive values of these epigenetic events in cancer of the oral cavity and pharynx. Our results suggest that *MGMT *hypermethylation is one of the prognostic factors in oral and pharyngeal cancer patients' survival; similar findings were reported for head and neck patients in general by some authors [21,23] but not by others [24].

Among the strengths of our study is the well characterized population of oral and pharyngeal cancers, which has detailed baseline epidemiological data on risk factors including HPV testing and complete therapy information, as well as complete follow-up data. In addition, a comparison between tissue and peripheral blood methylation from the same patient was possible because of the availability of both biological samples. A possible weakness of this study is that although this is the largest oral and pharyngeal cancer study using follow up data in relation to promoter methylation, the sample size is still relatively small. Additionally, the tumor samples used were not microdissected, leaving open the possibility of contamination from surrounding normal tissue and stroma, although previous studies have demonstrated that *MGMT *is not generally methylated in normal head and neck tissue [31,32].

In our cohort of patients, a methylation frequency similar to that reported in the literature was observed for *MGMT *(25-52%) and for *RASSF1 *(0-8%), while a lower value for *CDKN2A *(23-67%) was observed [11]. It should be noted that the studies recently reviewed [11] were conducted on small populations from different ethnic backgrounds. It is possible that environmental exposures as well as distinct genetic pathways to cancer development may explain the wide range of frequencies reported in the literature [33]. We also analyzed the tumor methylation pattern of the combination of the three genes under study. We observed that in roughly half of the tumors, none of the three genes were methylated, while in a small proportion of the tissues (roughly 10%), at least two of the genes under study were hypermethylated. This suggests that different carcinogenetic pathways may be present in tumors that are otherwise similar for histology and location. Unfortunately, the small size of the study does not allow any further speculation on this issue. *CDKN2A *was not methylated in any of the HPV-positive samples. This observation is consistent with the hypothesis that HPV-associated head and neck cancers have a distinct etiology, since HPV-positive tumors are more likely to over-express p16 [34].

No direct association between promoter hypermethylation in *MGMT *or *RASSF1 *and age, sex, smoking, alcohol drinking, or tumor stage was observed, in agreement with recently reported data [16,17,35]. A possible association between drinking and stage with methylation of *CDKN2A *was observed, although small sample size requires confirmation of this result. The comparison of the findings in the tumor tissue and in the peripheral blood from the same patient showed no *RASSF1 *or *CDKN2A *methylation in any of the peripheral blood samples, and only one sample positive for *MGMT *methylation; a T4N0 M0 (stage IVa) squamous cancer of the oral cavity. Although no metastasis was identified at diagnosis, it is conceivable that the positive blood samples were due to circulating tumor cells from the advanced stage tumor. These results indicate that hypermethylation is tumor specific and is not a general characteristic of other tissues or cell types of the individual at risk for tumor recurrence.

Epigenetic changes in cancer, more recently referred to as the cancer epigenome, have traditionally been evaluated by measuring the status of CpG island cytosine methylation of a particular gene such as *MGMT *[3] using Methylation-Specific PCR (MSP-PCR). MSP-PCR is a well-established, straightforward and rapid PCR-based method for analysis of promoter methylation and gene silencing. However, MSP-PCR is not quantitative and therefore it was of interest to determine whether variable amounts of *MGMT *promoter methylation could be detected in these tumors as a function of biological characteristics of the tumor. To assess this, a quantitative pyrosequencing methylation assay was employed. The tumors found to be positively methylated by methylation-specific PCR indeed showed variable methylation levels ranging from 0.6% to 46.6% with a median level of 6.9%. This is in agreement with the findings of Shaw and colleagues [29], who report *MGMT *percent methylation ranging from 0.0%-45% among 37 head and neck tumor samples. The association between degree of *MGMT *hypermethylation and oral and pharyngeal cancer survival has not been reported before to our knowledge. Indirect evidence in support of our findings comes from previous reports on a negative correlation between MGMT mRNA levels and percent promoter methylation in head and neck tumors [26,36].

## Conclusion

We evaluated 88 primary oral and pharyngeal tumors for methylation of the promoter for the DNA repair gene *MGMT *and the tumor suppressor genes *CDKN2A and RASSF1 *using methylation-specific PCR. Further, quantitative *MGMT *methylation was assessed and these results were then correlated with survival and tumor recurrence. *MGMT *promoter methylation was significantly associated with poorer overall and disease-free survival and a significant trend with the amount of *MGMT *methylation and survival and recurrence was observed (p_trend _= 0.002 and 0.001 respectively). These results implicate *MGMT *promoter methylation as a possible biomarker for oral and pharyngeal cancer prognosis. It is therefore conceivable that as the degree of methylation in head and neck cancers increases, genomic stability declines as a result of decreasing MGMT expression, thus leading to poorer prognosis and tumor recurrence. Further, the altered DNA methylation landscape of the cancer epigenome is not limited to promoter hypermethylation of select genes but also includes global hypomethylation as a prelude to oncogene activation and genome instability [7-9]. The critical role of MGMT in DNA repair suggests that defective DNA repair may be correlative in the observed association between *MGMT *promoter methylation and tumor recurrence. We therefore suggest that follow-up studies include (*i*) further quantitative methylation analysis, such as the MethyLight [37-42] or pyrosequencing assay [26,27], (*ii*) a measurement of global methylation status in promoters and non-promoter regions [43] and (*iii*) a detailed analysis of downstream DNA repair genes regulated by methylation such as *MLH1*, *MSH2*, *ATM *etc. in an effort to identify a DNA repair gene methylation signature that may be responsive to patient outcome.

## List of abbreviations

MGMT: O^6^-methylguanine-DNA methyltransferase; MSP-PCR: Methylation-Specific PCR; HPV: human papillomavirus.

## Competing interests

The authors declare that they have no competing interests.

## Authors' contributions

XHW carried out the MGMT MSP-PCR reactions and analysis. XHW and ARB carried out the MGMT pyrosequencing analysis. CR carried out the RASSF1 and CDKN2A MSP-PCR reactions and analysis, participated in the preparation of the manuscript and performed the data and statistical analysis. JC and SL made substantial contributions in data acquisition, statistical analysis and helped in manuscript and figure preparation. SMG provided the purified DNA samples. ET, SG and RWS wrote the manuscript and participated in study and experimental design. All authors have read and approved the final manuscript.

## Pre-publication history

The pre-publication history for this paper can be accessed here:

http://www.biomedcentral.com/1471-2407/9/354/prepub
